# Genotype-expression interactions for *BDNF* across human brain regions

**DOI:** 10.1186/s12864-021-07525-1

**Published:** 2021-03-23

**Authors:** Patrick Devlin, Xueyuan Cao, Ansley Grimes Stanfill

**Affiliations:** 1 Department of Anatomy and Neurobiology, College of Graduate Health Sciences, University of Tennessee Health Science Center, 920 Madison Ave. #807, Memphis, TN 38163 USA; 2 Department of Acute and Tertiary Care, College of Nursing, University of Tennessee Health Science Center, 874 Union Ave. #120J, Memphis, TN 38163 USA

**Keywords:** BDNF, Genetic expression, Brain, Human subjects, Cognitive disorders

## Abstract

**Background:**

Genetic variations in brain-derived neurotrophic factor (*BDNF*) are associated with various psychiatric disorders including depression, obsessive-compulsive disorder, substance use disorders, and schizophrenia; altered gene expression triggered by these genetic variants may serve to create these phenotypes. But genotype-expression interactions for this gene have not been well-studied across brain regions relevant for psychiatric disorders.

**Results:**

At false discovery rate (FDR) of 10% (q < 0.1), a total of 61 SNPs were associated with *BDNF* expression in cerebellum (*n* = 209), 55 SNPs in cortex (*n* = 205), 48 SNPs in nucleus accumbens (*n* = 202), 47 SNPs in caudate (*n* = 194), and 58 SNPs in cerebellar hemisphere (*n* = 175). We identified a set of 30 SNPs in 2 haplotype blocks that were associated with alterations in expression for each of these 5 regions. The first haplotype block included variants associated in the literature with panic disorders (rs16917204), addiction (rs11030104), bipolar disorder (rs16917237/rs2049045), and obsessive-compulsive disorder (rs6265). Likewise, variants in the second haplotype block have been previously associated with disorders such as nicotine addiction, major depressive disorder (rs988748), and epilepsy (rs6484320/rs7103411).

**Conclusions:**

This work supports the association of variants within *BDNF* for expression changes in these key brain regions that may contribute to common behavioral phenotypes for disorders of compulsion, impulsivity, and addiction. These SNPs should be further investigated as possible therapeutic and diagnostic targets to aid in management of these and other psychiatric disorders.

**Supplementary Information:**

The online version contains supplementary material available at 10.1186/s12864-021-07525-1.

## Background

Brain-derived neurotrophic factor (gene symbol: *BDNF*) is a widely expressed protein in the nervous tissues of the brain and spinal cord, as it is responsible for the growth, maintenance, and maturation of nerve cells [1]. This protein is classified as a neurotrophin due to the important role it plays in regulating neuronal function and development, done so through highly regulated expression that ensures correct cell-to-cell communication and viability. The BDNF protein and its receptor, tropomyosin receptor kinase B (TrkB), are involved in several intracellular signaling pathways including the phospholipase Cg (PLCg), phosphoinositide 3-kinase (PI3K), and mitogen-activated protein kinase/extracellular signal-regulated protein kinase (MAPK/ERK) pathways [2]. Each of these pathways is important for neuronal signaling activities that will vary by expression across brain regions, including neuroregeneration, neurosynaptic plasticity, memory formation, and regulation of cognitive functions [3, 4]. Any significant change in the amount of expression can influence this signaling and thus result in downstream behavioral effects.

Given these broad signaling effects, it is not surprising that altered *BDNF* expression has also been implicated in development of neuropsychiatric disorders such as depression, obsessive-compulsive disorder, substance use disorders, and schizophrenia [5]. Indeed, alterations in *BDNF* peripheral blood (leukocyte) gene expression and protein levels have been shown to be a significant biomarker for many of these disorders [6, 7]. Genetic variations within *BDNF* may induce inappropriate expression and result in breakdown of proper cell signaling, and several variants in this gene have similarly been associated with various neuropsychiatric disorders [8, 9]. Conversely, restoration to normal homeostatic conditions has been shown to improve the symptoms caused by these conditions, as occurs when BDNF-targeted neurotrophic pharmaceuticals are given to patients [10].

However, *BDNF* expression and function are highly tissue-dependent. Due to the important regulatory role it plays, this protein can be found in most areas of the brain relevant to psychiatric disorders, including the cortex, cerebellum, caudate, hippocampus, and others. Expression alterations will vary by disease and by brain region/location [5]. For instance, in schizophrenia, prefrontal cortex BDNF protein levels were found to be significantly lower in patients versus healthy controls [11–13], while decreases in BDNF levels in the temporal cortex and occipital cortex, and increases in the parietal cortex and frontal cortex, were found in postmortem samples [14]. Despite these findings, and other evidence that expression changes are associated with psychiatric behavioral profiles, it is still unclear how genotypic variation is associated with expression of *BDNF* across these relevant human brain regions. Here, we will address this gap by using the Genotype-Tissue Expression (GTEx) database to determine the associations of variations in *BDNF* with expression across five brain regions relevant to neuropsychiatric conditions. Understanding these differences will allow better comprehension of regional variations in expression and how genetic variation contributes to differential expression by brain region. This information is the first step toward future work in *BDNF* signal regulation and the development of related therapeutics.

## Results

### Human subject and tissue sample data

Expression levels from a total of 985 tissue samples from 300 unique subjects were matched to genetic sequencing data for *BDNF* for our 5 brain regions of interest (Table 1). Note that not every person donated samples from every brain region, leading to a discrepancy in the table between the numbers of tissue samples that were available for each region. Summary data for the four demographic factors of our included subjects (age, sex, race, and BMI) are provided in Table 2.

**Table 1:** *BDNF* expression data by tissue

Brain Tissue Region	Number of subjects	Median (TPM)	Fraction (0 TPM)	Subjects (> 0 TPM)
Cerebellum	209	24	0.177	172
Cortex	205	21	0.278	148
Nucleus accumbens	202	17	0.2772	146
Caudate	194	18.5	0.2629	143
Cerebellar Hemisphere	175	25	0.24	133

**Table 2:** Demographics of human subjects (*N* = 300)

Variables	Level	n or mean (% or range)
Sex	Female	86 (28.7%)
	Male	214 (71.3%)
Race	American Indian or Alaska Native	1 (0.3%)
	Asian	1 (0.3%)
	Black	26 (8.7%)
	White	272 (90.7%)
Age		60.5 (53–65)
BMI		27.34 (24.4–30.9)

### Association between BDNF expression by demographics

Age, sex, and BMI were not associated with *BDNF* expression for any of our five brain regions of interest in this sample (*p* > 0.08; Table 3). Race was associated with *BDNF* expression in the caudate tissue samples (*p* = 0.01), but this association was not found for the remaining regions.

**Table 3:** Association of *BDNF* expression with demographic factors

Region	Demographic		n	Median_IQR	*p*-value
Cerebellum	Sex	Female	48 (27.9%)	30 (13.75 ~ 45.25)	0.97
		Male	124 (72.1%)	30.5 (9.75 ~ 50.25)	
	Race	Black	17 (9.9%)	18 (13 ~ 33)	0.13
		White	154 (89.5%)	32 (12.25 ~ 50.75)	
	Age		172	−0.03	0.70
	BMI		172	−0.0063	0.93
Cortex	Sex	Female	49 (33.1%)	27 (14 ~ 39)	0.18
		Male	99 (66.9%)	33 (17.5 ~ 46)	
	Race	Black	17 (11.5%)	22 (4 ~ 38)	0.08
		White	130 (87.8%)	31.5 (18.25 ~ 45)	
	Age		148	−0.0238	0.77
	BMI		148	-9e-04	0.99
Nucleus accumbens	Sex	Female	42 (28.8%)	25.5 (12.25 ~ 42.75)	0.90
		Male	104 (71.2%)	28 (15 ~ 41.25)	
	Race	Black	13 (9%)	20 (3 ~ 35)	0.14
		White	131 (90.3%)	28 (15 ~ 43)	
	Age		146	0.0486	0.56
	BMI		146	0.0778	0.35
Caudate	Sex	Female	38 (26.6%)	33 (18.25 ~ 55)	0.14
		Male	105 (73.4%)	28 (14 ~ 40)	
	Race	Black	14 (9.9%)	15.5 (2.25 ~ 26.5)	0.01
		White	127 (89.4%)	33 (17 ~ 46)	
	Age		143	0.031	0.71
	BMI		143	0.0031	0.97
Cerebellar Hemisphere	Sex	Female	37 (27.8%)	44 (16 ~ 70)	0.79
		Male	96 (72.2%)	42 (18.75 ~ 69.5)	
	Race	Black	11 (8.3%)	25 (16 ~ 40)	0.09
		White	121 (91%)	45 (19 ~ 72)	
	Age		133	−0.0192	0.83
	BMI		133	0.1449	0.10

### Differential BDNF expression between brain regions

**Fig. 1:**
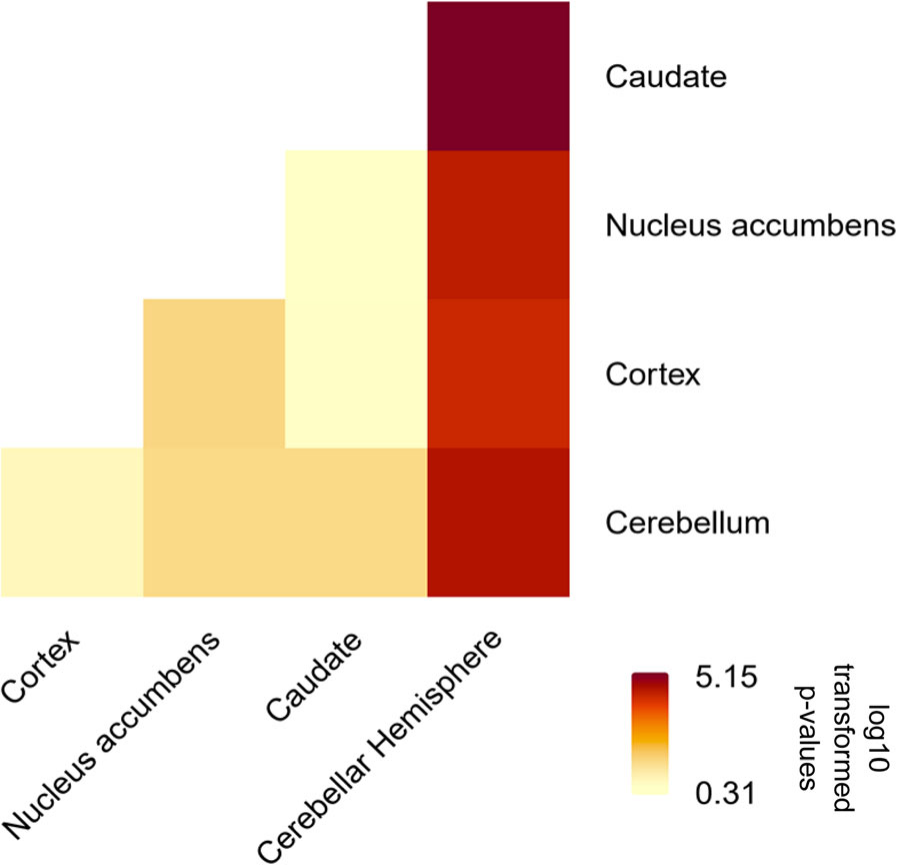
Heatmap of BDNF differential expression among five brain regions. The *p*-values of pairwise signed rank tests were log10 transformed, negated and plotted. A value greater than 1.3 indicates the *p*-value less than 0.05 and significant differential expression

Next, we investigated the pairwise differential expression among the brain regions of interest. The expression of *BDNF* in cerebellar hemisphere was significantly higher than that in cerebellum, cortex, caudate and nucleus accumbens (*p* < 0.0001, Table 4**;** Fig. 1)**.**

**Table 4:** BDNF differential expression among five brain tissues

Tissues	Cortex	Nucleus accumbens	Caudate	Cerebellar Hemisphere
Cerebellum	0.209094	0.036692	0.036883	3.1e-05
Cortex		0.033255	0.370596	6.9e-05
Nucleus accumbens			0.491501	4.6e-05
Caudate				7e-06

Note that the *p*-value of the signed rank test compares median expression between two regions.

### Genotype-expression in brain tissue

**Fig. 2:**
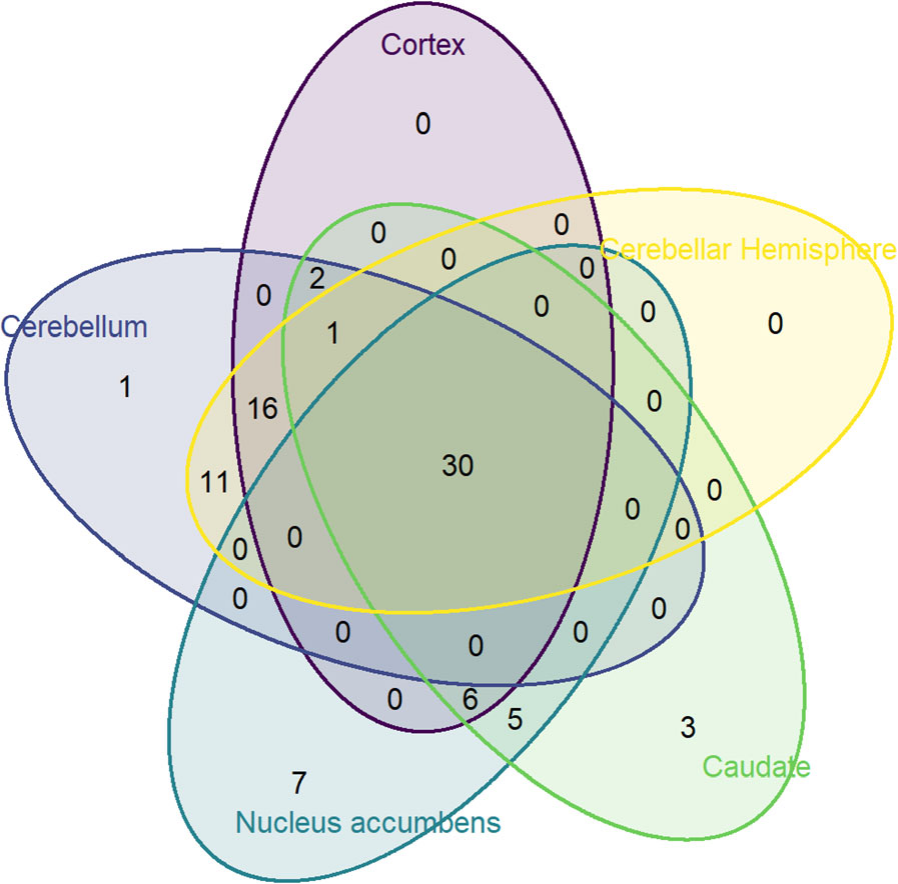
Venn diagram of Cis-expression Quantitative Trait Loci (eQTL) of BDNF across five human brain regions

**Fig. 3:**
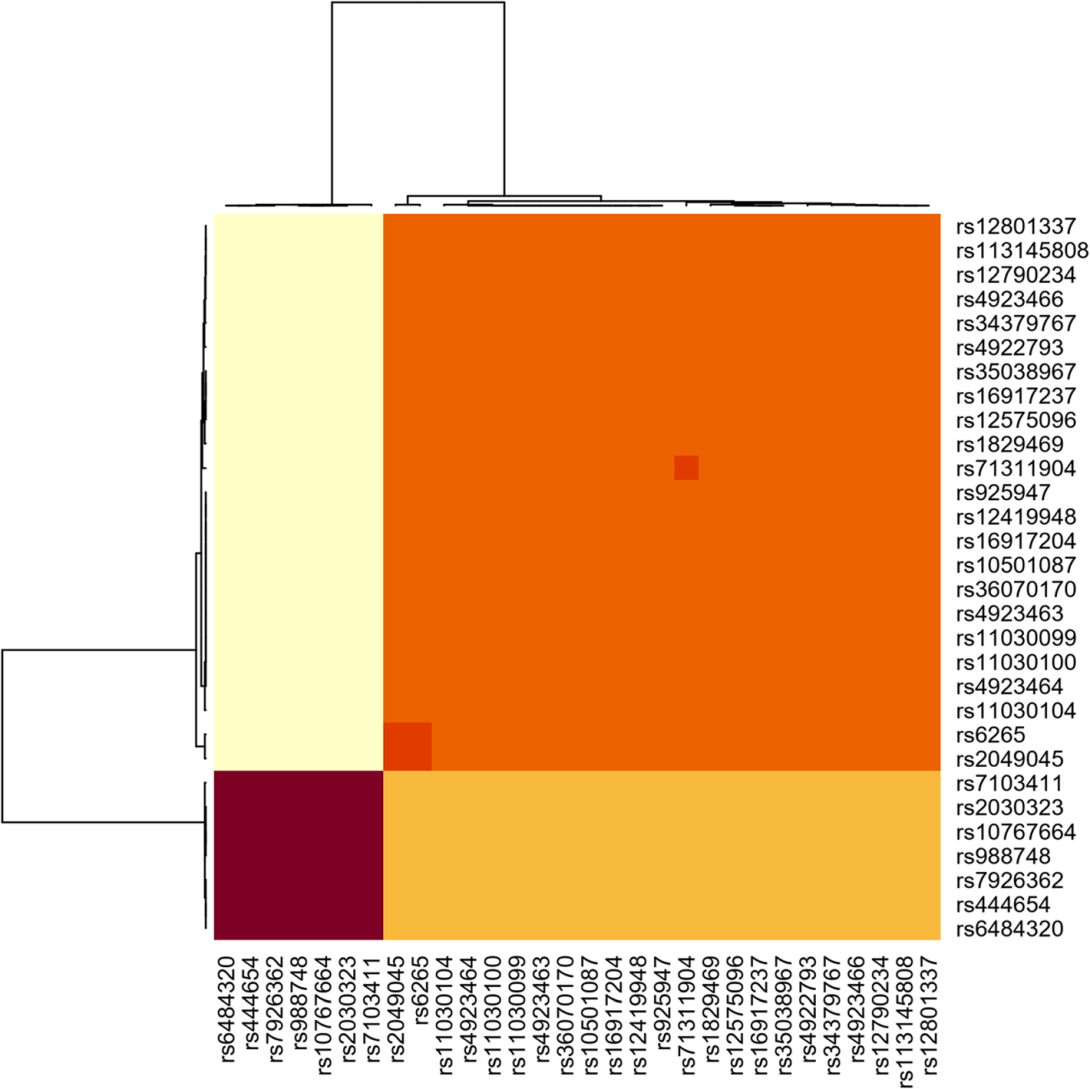
Haplotype groups of significant SNPs shared across the brain regions of interest

A total of 61 SNPs in *BDNF* were associated with expression in the cerebellum, 55 SNPs in cortex, 48 SNPs in nucleus accumbens, 47 SNPs in caudate, and 58 SNPs in cerebellar hemisphere at FDR level of 0.1 (Supplementary Table 1). Thirty of these SNPs were shared and thus significant for *BDNF* expression across all five brain regions (*p* < 0.000015, Fig. 2). These SNPs were aggregated into two main haplotype blocks, with eight SNPs (rs16917204, rs11030104, rs7103411, rs16917237, rs6484320, rs988748, rs2049045, rs6265) associated in the literature with various psychiatric disorders (Fig. 3; Table 5). Table 6 provides the detailed direction and strength of associations measured by Spearman correlation across the 5 brain regions for the 30 SNPs.

**Table 5:** Clinically relevant SNPs associated with *BDNF* expression across brain regions of interest

SNP rsID	1000 Genome Frequency	Location	Associated Disorder or Phenotype
rs16917204	C = 0.2298	Intron, gene body, Chromosome 11, Position: 27646808	• Methamphetamine abuse [15]
			• Panic disorders [16]
			• Alzheimers Disease [17]
			• Schizophrenia [18]
rs6265	T = 0.2013	Exon, gene body, Chromosome 11, Position: 27658369	• Major depressive disorder [19]
			• Bipolar disorder [20]
			• Obsessive-compulsive disorder [21]
			• Alzheimer’s disease [22]
rs11030104	G = 0.2226	Intron, gene body, Chromosome 11, Position: 27662970	• Increased BMI [23]
			• BMI and Smoking [24]
			• Alzheimer’s disease [22]
rs7103411	C = 0.2470	Intron, gene body, Chromosome 11, Position: 27678578	• Epilepsy [25]
rs16917237	T = 0.2214	Intron, gene body, Chromosome 11, Position: 27680836	• Eating disorders, increased BMI [26]
			• Bipolar disorder [27]
rs6484320	T = 0.2468	Intron, gene body, Chromosome 11, Position: 27681641	• Epilepsy (in Fragile x-syndrome) [28]
			• Smoking and nicotine addiction [29]
rs988748	C = 0.2430	Intron, gene body, Chromosome 11, Position: 27703198	• Major depressive disorder [19]
			• Smoking and nicotine addiction [29]
rs2049045	C = 0.0629	Intron, gene body, Chromosome 11, Position: 27672694	• Alzheimer’s disease-related depression [30]
			• Bipolar disorder [31]
			• Alzheimer’s disease [22]

**Table 6:** The association of 30 SNPs with BDNF expression in each of the five regions

			ALT Freq	Cerebellum		Cortex		Nucleus accumbens		Caudate		Cerebellar Hemisphere	
dbSNP_ID	REF	ALT		Corr_r	***P*** value	Corr_r	***P*** value	Corr_r	***P*** value	Corr_r	***P*** value	Corr_r	***P*** value
rs2049045	G	C	0.1531	0.5676	4.73E-16	0.5758	1.93E-14	0.4088	3.02E-07	0.4473	2.14E-08	0.5266	7.51E-11
rs6265	C	T	0.1555	0.584	4.12E-17	0.5974	1.10E-15	0.4084	3.09E-07	0.4666	4.27E-09	0.5475	9.10E-12
rs1829469	A	G	0.1675	0.5391	3.97E-14	0.4754	1.17E-09	0.3581	9.10E-06	0.3918	1.43E-06	0.5226	1.30E-10
rs16917237	G	T	0.1699	0.5185	3.21E-13	0.4789	7.42E-10	0.3506	1.44E-05	0.4025	6.23E-07	0.5127	2.81E-10
rs35038967	T	A	0.1699	0.5185	3.21E-13	0.4789	7.42E-10	0.3506	1.44E-05	0.4025	6.23E-07	0.5127	2.81E-10
rs12575096	A	G	0.1699	0.5185	3.21E-13	0.4789	7.42E-10	0.3506	1.44E-05	0.4025	6.23E-07	0.5127	2.81E-10
rs11030104	A	G	0.1722	0.5452	1.05E-14	0.4978	1.21E-10	0.3672	5.12E-06	0.3867	1.84E-06	0.5254	8.45E-11
rs71311904	C	CCATTT	0.1722	0.4743	4.96E-11	0.455	6.28E-09	0.3561	1.03E-05	0.4131	2.93E-07	0.4562	3.43E-08
rs34379767	G	A	0.1731	0.5075	1.20E-12	0.476	1.11E-09	0.3694	4.47E-06	0.4268	1.18E-07	0.4994	9.39E-10
rs4923466	C	A	0.1731	0.5086	1.22E-12	0.4866	4.13E-10	0.3694	4.47E-06	0.4334	6.39E-08	0.4994	9.39E-10
rs12419948	T	A	0.1746	0.5518	4.31E-15	0.5069	4.90E-11	0.3672	5.12E-06	0.3773	3.39E-06	0.5367	2.74E-11
rs925947	G	T	0.1746	0.5518	4.31E-15	0.5069	4.90E-11	0.3672	5.12E-06	0.3773	3.39E-06	0.5367	2.74E-11
rs16917204	G	C	0.1746	0.5518	4.31E-15	0.5069	4.90E-11	0.3672	5.12E-06	0.3773	3.39E-06	0.5367	2.74E-11
rs10501087	T	C	0.1746	0.5518	4.31E-15	0.5069	4.90E-11	0.3672	5.12E-06	0.3773	3.39E-06	0.5367	2.74E-11
rs36070170	A	AT	0.1746	0.5518	4.31E-15	0.5069	4.90E-11	0.3672	5.12E-06	0.3773	3.39E-06	0.5367	2.74E-11
rs4923463	A	G	0.1746	0.5518	4.31E-15	0.5069	4.90E-11	0.3672	5.12E-06	0.3773	3.39E-06	0.5367	2.74E-11
rs11030099	C	A	0.1746	0.5518	4.31E-15	0.5069	4.90E-11	0.3672	5.12E-06	0.3773	3.39E-06	0.5367	2.74E-11
rs11030100	G	T	0.1746	0.5518	4.31E-15	0.5069	4.90E-11	0.3672	5.12E-06	0.3773	3.39E-06	0.5367	2.74E-11
rs4923464	C	T	0.1746	0.5518	4.31E-15	0.5069	4.90E-11	0.3672	5.12E-06	0.3764	3.60E-06	0.5367	2.74E-11
rs113145808	T	C	0.1746	0.5075	1.20E-12	0.4758	9.88E-10	0.3694	4.47E-06	0.4334	6.39E-08	0.4994	9.39E-10
rs12801337	G	A	0.1746	0.5075	1.20E-12	0.4758	9.88E-10	0.3694	4.47E-06	0.4334	6.39E-08	0.4994	9.39E-10
rs12790234	A	G	0.1746	0.5075	1.20E-12	0.4758	9.88E-10	0.3694	4.47E-06	0.4334	6.39E-08	0.4994	9.39E-10
rs4922793	A	G	0.1755	0.5019	2.69E-12	0.4918	2.53E-10	0.3694	4.47E-06	0.4451	2.86E-08	0.4782	5.83E-09
rs988748	C	G	0.8182	−0.4983	3.49E-12	−0.4896	2.71E-10	−0.3755	3.02E-06	−0.4334	6.39E-08	−0.476	7.02E-09
rs10767664	T	A	0.8182	−0.4983	3.49E-12	−0.4896	2.71E-10	−0.3755	3.02E-06	−0.4334	6.39E-08	−0.476	7.02E-09
rs2030323	A	C	0.8182	−0.4983	3.49E-12	−0.4896	2.71E-10	−0.3755	3.02E-06	−0.4334	6.39E-08	−0.476	7.02E-09
rs6484320	T	A	0.8206	− 0.514	5.51E-13	− 0.4896	2.71E-10	− 0.3755	3.02E-06	− 0.4334	6.39E-08	− 0.497	1.16E-09
rs444654	T	G	0.8206	−0.514	5.51E-13	−0.4896	2.71E-10	−0.3755	3.02E-06	−0.4334	6.39E-08	−0.497	1.16E-09
rs7926362	A	C	0.8221	−0.5099	1.06E-12	−0.4896	2.71E-10	−0.3768	3.00E-06	−0.4352	6.21E-08	−0.497	1.16E-09
rs7103411	C	T	0.8230	−0.5098	9.16E-13	−0.4896	2.71E-10	−0.3847	1.63E-06	−0.4334	6.39E-08	−0.48	5.03E-09

Note: *REF* Reference allele; *ALT* Alternative allele; *Freq* Frequency; *Corr_r* Spearman correlation estimates, Correlations and *p*-values across some SNPs are identical due to presence within a haplotype block

## Discussion

Our results are some of the first to demonstrate genotype-expression interactions across multiple human brain regions relevant for neuropsychiatric conditions. Our work suggests the physiological (here, gene expression) result of genetic *BDNF* variation that may contribute to specific psychiatric disorders in symptomatology and behavioral phenotypes. In turn, future investigations of these SNPs will lead to a better understanding of the complexity of BDNF protein signaling in the human brain and will inform future therapeutics in this area.

### Association between BDNF expression and clinical and demographic factors

Surprisingly, the GTEx sample did not demonstrate a relationship between the four available demographic factors and expression in these brain regions. *BDNF* expression has been shown in other research to vary by sex, possibly due to sexual dimorphism in hormonal status, enzymatic activities, and body weight (BMI) as well as fat/lean mass composition differences by sex [32]. For example, sex differences in estrogen regulation have been shown to alter *BDNF* gene expression. Females have been shown to have a lower baseline *BDNF* gene expression than males in the cortex as well as CA1 regions of the hippocampus and dentate gyrus [33, 34]. We did not observe such significant difference in any of the regions (*p* > 0.08; Table 3). While the samples available in this database are extensive, such hormonal and enzymatic contributors may be more influential in an in-vivo system than in the post-mortem tissue samples we are analyzing here and could potentially lead to this lack of association.

Increased age has also been strongly associated with a decrease in *BDNF* expression in the cortex, potentially from neuronal death during the aging process [35, 36]. Similar changes have been found in hippocampal volume and *BDNF* expression [37]. However, our work here did not demonstrate such effects, possibly due to the limited age range of our subjects between 53 and 65 years old, often before such volumetric loss becomes significant (Table 3). Although research has shown that significant alteration of gene expression precedes the psychiatric phenotype and often occurs in the prenatal stages [38], it is one limitation of our dataset that data from prenatal subjects are not available .

### Differential expression of BDNF across brain regions

Our results demonstrate that there are significant variations in *BDNF* expression across each of our brain regions of interest. Expression of *BDNF* in the cerebellar hemisphere was significantly higher than the cerebellum (*p* = 3.1e-05), cortex (*p* = 6.9e-05), nucleus accumbens (*p* = 4.6e-05), and caudate (*p* = 7e-06) (Table 4; Fig. 1). This suggests that the signaling activities of *BDNF* may be more robust in these areas of the brain, creating functional changes in these areas. Although there is a dearth of literature to support such associations in human brain regions, our results are consistent with findings in mouse models, with higher levels of *BDNF* mRNA being found in the cerebral cortex and cerebellum [39].

Further, there is a logic to these findings, as for example, the cerebellum requires greater *BDNF* expression to aid in transport during complex functions such as motor coordination and information-processing. Clinically, dysfunction in these brain regions is associated with various neuropsychiatric disorders. The large amounts of *BDNF* localization in these areas demonstrates their importance in signaling to maintain neuropsychiatric functions. For instance, decreased expression of *BDNF* occurs in the prefrontal cortex and nucleus accumbens (NAc) of human patients with major depression [40, 41], while bipolar disorder has been associated with decreased *BDNF* levels and grey matter reductions in various subcortical structures implicated in emotional processing [42–45]. A greater understanding of the clinical implications of these differences may provide unique insight to the overall role of *BDNF* in the neurological system.

### SNPs associated with expression across brains regions

While there are region specific genotype-expression associations, there are also commonalities in genotype-expression that are shared across all five regions, a finding which suggests that different areas of the brain may share common signaling pathways and harness together common expression changes that are associated with neuropsychiatric phenotypes. While many of our shared SNPs showed little clinical relevance in the literature to date, several were shown to play a role in the development of various types of psychiatric disorders—from cognitive based conditions, to bipolar disorder, depression, and epilepsy. Several others were also shown to play a role in addictive behaviors (Table 5). For example, rs16917204 has been found to be associated with panic disorder, Alzheimer’s, and schizophrenia [16–18]. But the influence of a particular SNP upon phenotype is dependent on the expression alteration and the influence on the neurosignaling behavior of that local region upon other regions of the brain.

This phenomenon is clearly illustrated by the effect of Val66Met (rs6265), a common polymorphism in the *BDNF* pathway, which was found to have genotype-expression effects across our most significant five brain regions. In both post-traumatic stress disorder and bipolar disorder, rs6265 is a locus for trauma-induced epigenetic regulation, which alters expression to reduce *BDNF* production, and is thus a contributor to the onset of both illnesses through the mediation of the neurotrophic receptor tyrosine kinase 2 (*NTRK2*) [46–48].

Indeed, rs6265 is one of the most widely studied and understood SNPs responsible for *BDNF* expression changes. In this SNP, the substitution of a valine to methionine at codon 66 affects gene expression by resulting in the dysregulation of microRNA (such as miR-146b), which in turn affects downstream mRNA levels resulting in altered protein expression [49, 50]. This change in protein expression effects integral processes such as synaptic plasticity which results in many of the cognitive orders associated with this SNP [51, 52]. Understanding the underlying processes responsible for the phenotypic presentation of observed disorders can better provide insight into the role of specific SNPs and inform possible targets for medical interventions in humans.

Others of our SNPs of interest create common phenotypes in a different manner. As an example, the SNPs rs7103411 and rs6484320 are both located on Chromosome 11, both intronic, and both associated with epilepsy (Table 5). The SNP rs7103411 is significantly associated with cryptogenic and symptomatic epilepsy [25], while rs6484320 is specifically associated with epilepsy occurring in Fragile X syndrome [28]. However, the mechanism of action to create the common seizure phenotype is different, as rs6484320 does not itself directly affect *BDNF* expression through its receptors but instead plays this role through a linkage disequilibrium with Val66Met [53]. The SNP rs7103411 directly affects expression through receptor disruption and influences the abnormal signaling behavior seen in epilepsy [25].

The majority of our SNPs are found on the non-coding regions of the gene body, which may affect splicing or gene expression as cis-regulatory elements. However, these SNPs have been demonstrated to alter transcription or affect splicing of the pre-RNA transcript [54, 55]. Many “silent” SNPs can cause the generation of proteins with the same amino acid sequences, but which have different structural and functional properties by changing conformation and protein activity/substrate specificity [56, 57]. These phenomenon have been illustrated in the associations of major depressive disorder with rs988748, and in schizophrenia rs16917204 [53, 58].

Our SNPs can also interact in a synergistic manner to create the observed *BDNF* expression changes, as which occurs in Alzheimer’s disease. The SNPs rs11030104 and rs2049045 are found between exons 6 and 7. On the other hand, SNP rs6265, previously discussed, is found on exon 8. One study found that the haplotype of rs6265, rs2049045, and rs11030104 were all significant in Apolipoprotein E (*APOE4*) non-carriers, and the collective disruption of transcription in these coding regions, caused by the shared effects of these polymorphisms, results in decreased *BDNF* expression [22]. Thus, the combined effect of these three SNPs and diplotypes increases the risk for Alzheimer’s disease development in APOE 4 non-carriers [22].

Along with previous research, our results here have highlighted that *BDNF* expression can differ significantly across various brain regions. This expression is influenced by several genetic phenomenon, which includes the influence of shared SNPs. While we don’t have psychiatric phenotype data available directly from the GTEx database, we were able to show that these SNPs can influence underlying biological functions. Ultimately, these alterations can result neuropsychiatric conditions.

## Conclusions

Here, using the GTEx database, we have identified significant genotype-expression interactions for *BDNF* across five human brain regions (cerebellum, cortex, nucleus accumbens, caudate, and cerebellar hemisphere). Although the magnitude of these alterations differed across the tested regions, there were 30 different SNPs in 2 haplotype blocks that were associated with alterations in expression across these 5 areas. Moving forward, these identified targets can be used in the development of possible treatments and therapies that aim to restore proper *BDNF* signaling. Ultimately, these shared SNPs can also provide us with possible targets for treatment in neuropsychiatric condition disorders of compulsion, impulsivity, and addiction, including depression, obsessive-compulsive disorder, substance use disorders, and schizophrenia.

## Methods

### Genotype-tissue expression (GTEx) database

Supported by the National Institutes of Health, the GTEx dataset contains phenotypic and molecular data from around 1000 adult human subjects across 54 tissue types. These tissues range from blood and lung samples to brain tissue. A limited set of clinical and demographic factors are also included. Users can browse the database using portal tools to gather some information on their gene(s) of interest, variant information, and tissue expression quantitative trait loci (eQTL) data (https://gtexportal.org/home/). Here, we applied for access to an additional restricted dataset that includes RNA-seq and DNA sequencing information through the National Center for Biotechnology Information’s Database of Genotypes and Phenotypes (dbGaP;https://dbgap.ncbi.nlm.nih.gov/). After obtaining access, we limited our investigations to available single-nucleotide polymorphism (SNP) data and *BDNF* expression across five brain regions relevant to neuropsychiatric conditions, considering only those which had non-zero expression (measured as Transcripts Per kilobase [TPM]) for *BDNF*: cerebellum, cortex, nucleus accumbens, caudate, and cerebellar hemisphere. Of note, cerebellum and cerebellar hemisphere represent samples that were collected under different collection and processing procedures. To clarify, the “cerebellar hemisphere” region includes both the right and left cerebellar hemispheres that were sampled at Miami Brain Bank and preserved as fresh frozen tissue. The data obtained from the “cerebellum” region is made up of samples of the right cerebellum sampled at the main donor collection site and preserved in PAXgene fixative. Due to the nature of these sample collection procedures, this measures the differences between the right and left cerebellar hemispheres. For transparency and replicability, we have used the labeling as used in the GTEx database. All other brain regions were specifically sampled at Miami Brain Bank and preserved as fresh frozen tissue (https://www.gtexportal.org/home/samplingSitePage).

After these regions were selected, we investigated the association of *BDNF* genotype with expression, associations between expression and four clinical and demographic factors also known to be important for the development of neuropsychiatric conditions: age, sex, race, and body mass index (BMI) [59–63]. Then we assessed for SNPs that were associated with expression variations across all 5 brain regions.

### Data analysis

Summary statistics were collected along with the median expression of *BDNF* in each region. The association of *BDNF* expression with sex and race were tested in each region using Kruskal-Wallis tests. Wilcoxon signed-rank test was used to test equality of median expression level of *BDNF* between two paired brain regions. Associations of *BDNF* expression with age, BMI, and number of alternative alleles of each of our SNPs of interest were measured for each region by Spearman correlation. We elected to use a conservative rank based test with false discovery rate (FDR) estimated by the Benjamini and Hochberg method, with a cutoff value was set to 0.1 (at most, 10% false positive) in each brain region [64]. All analyses were performed using R- 3.6.1.

## Supplementary Information

**Additional file 1.**

## Data Availability

Individual investigators seeking access to GTEx-controlled data must apply through the National Center for Biotechnology Information’s (NCBI) database of Genotypes and Phenotypes (dbGaP) at https://dbgap.ncbi.nlm.nih.gov/. The data used in this study were that included in the General Research Use set (phs000424.v8.p2.c1), NHGRI.

## Abbreviations

BDNF: Brain-derived neurotrophic factor
SNP: Single-nucleotide polymorphism
Val66Met: Rs6265 single-nucleotide polymorphism
mRNA: Messenger RNA
APOE4: Apolipoprotein E
