# Supplementary material for: Genotype-expression interactions for BDNF across human brain regions
Source: BMC Genomics. 2021 Mar 23;22:207. doi: 10.1186/s12864-021-07525-1 (PMC7989003; doi:10.1186/s12864-021-07525-1)
Supplement: Supplementary file 1 — Additional file 1. [file 12864_2021_7525_MOESM1_ESM.docx]

| **Supplementary Table 1.** BDNF Genotype-expression variants by tissue (detailed information) | | | | | | |
| --- | --- | --- | --- | --- | --- | --- |
| **Tissue** | **SNP** | **Alternative Allele Frequency** | **Call Rate** | **Spearman r** | ***p*-value** | **q-value** |
| Caudate (basal ganglia) | rs6265 | 0.157216 | 1 | 0.4666 | 4.27E-09 | 3.72E-07 |
| Caudate (basal ganglia) | rs2049045 | 0.154639 | 1 | 0.4473 | 2.14E-08 | 1.59E-06 |
| Caudate (basal ganglia) | rs4922793 | 0.176166 | 0.994845 | 0.4451 | 2.86E-08 | 2.09E-06 |
| Caudate (basal ganglia) | rs7926362 | 0.826425 | 0.994845 | -0.4352 | 6.21E-08 | 4.14E-06 |
| Caudate (basal ganglia) | rs7103411 | 0.824742 | 1 | -0.4334 | 6.39E-08 | 4.14E-06 |
| Caudate (basal ganglia) | rs6484320 | 0.824742 | 1 | -0.4334 | 6.39E-08 | 4.14E-06 |
| Caudate (basal ganglia) | rs444654 | 0.824742 | 1 | -0.4334 | 6.39E-08 | 4.14E-06 |
| Caudate (basal ganglia) | rs113145808 | 0.175258 | 1 | 0.4334 | 6.39E-08 | 4.14E-06 |
| Caudate (basal ganglia) | rs4923466 | 0.175258 | 1 | 0.4334 | 6.39E-08 | 4.14E-06 |
| Caudate (basal ganglia) | rs12801337 | 0.175258 | 1 | 0.4334 | 6.39E-08 | 4.14E-06 |
| Caudate (basal ganglia) | rs12790234 | 0.175258 | 1 | 0.4334 | 6.39E-08 | 4.14E-06 |
| Caudate (basal ganglia) | rs988748 | 0.822165 | 1 | -0.4334 | 6.39E-08 | 4.14E-06 |
| Caudate (basal ganglia) | rs10767664 | 0.822165 | 1 | -0.4334 | 6.39E-08 | 4.14E-06 |
| Caudate (basal ganglia) | rs2030323 | 0.822165 | 1 | -0.4334 | 6.39E-08 | 4.14E-06 |
| Caudate (basal ganglia) | rs34379767 | 0.173575 | 0.994845 | 0.4268 | 1.18E-07 | 7.34E-06 |
| Caudate (basal ganglia) | rs71311904 | 0.17268 | 1 | 0.4131 | 2.93E-07 | 1.65E-05 |
| Caudate (basal ganglia) | rs16917237 | 0.170103 | 1 | 0.4025 | 6.23E-07 | 3.40E-05 |
| Caudate (basal ganglia) | rs35038967 | 0.170103 | 1 | 0.4025 | 6.23E-07 | 3.40E-05 |
| Caudate (basal ganglia) | rs12575096 | 0.170103 | 1 | 0.4025 | 6.23E-07 | 3.40E-05 |
| Caudate (basal ganglia) | rs1829469 | 0.164063 | 0.989691 | 0.3918 | 1.43E-06 | 7.14E-05 |
| Caudate (basal ganglia) | rs11030104 | 0.17268 | 1 | 0.3867 | 1.84E-06 | 8.64E-05 |
| Caudate (basal ganglia) | rs12419948 | 0.17268 | 1 | 0.3773 | 3.39E-06 | 0.000138 |
| Caudate (basal ganglia) | rs925947 | 0.17268 | 1 | 0.3773 | 3.39E-06 | 0.000138 |
| Caudate (basal ganglia) | rs16917204 | 0.17268 | 1 | 0.3773 | 3.39E-06 | 0.000138 |
| Caudate (basal ganglia) | rs10501087 | 0.17268 | 1 | 0.3773 | 3.39E-06 | 0.000138 |
| Caudate (basal ganglia) | rs36070170 | 0.17268 | 1 | 0.3773 | 3.39E-06 | 0.000138 |
| Caudate (basal ganglia) | rs4923463 | 0.17268 | 1 | 0.3773 | 3.39E-06 | 0.000138 |
| Caudate (basal ganglia) | rs11030099 | 0.17268 | 1 | 0.3773 | 3.39E-06 | 0.000138 |
| Caudate (basal ganglia) | rs11030100 | 0.17268 | 1 | 0.3773 | 3.39E-06 | 0.000138 |
| Caudate (basal ganglia) | rs4923464 | 0.175258 | 1 | 0.3764 | 3.60E-06 | 0.000141 |
| Caudate (basal ganglia) | rs76069707 | 0.628866 | 1 | 0.3145 | 0.00013 | 0.002553 |
| Caudate (basal ganglia) | rs78500263 | 0.636598 | 1 | 0.3123 | 0.000146 | 0.002752 |
| Caudate (basal ganglia) | rs4517468 | 0.639896 | 0.994845 | 0.3123 | 0.000146 | 0.002752 |
| Caudate (basal ganglia) | rs2353487 | 0.636598 | 1 | 0.3123 | 0.000146 | 0.002752 |
| Caudate (basal ganglia) | rs10767659 | 0.637306 | 0.994845 | 0.3128 | 0.00015 | 0.002809 |
| Caudate (basal ganglia) | rs1519480 | 0.64433 | 1 | 0.2991 | 0.000285 | 0.004084 |
| Caudate (basal ganglia) | rs7481773 | 0.456186 | 1 | 0.2437 | 0.003353 | 0.028829 |
| Caudate (basal ganglia) | rs7127507 | 0.313472 | 0.994845 | -0.2404 | 0.003953 | 0.031784 |
| Caudate (basal ganglia) | rs1808124 | 0.688144 | 1 | 0.2367 | 0.004425 | 0.0348 |
| Caudate (basal ganglia) | rs1491851 | 0.494845 | 1 | 0.2323 | 0.005231 | 0.038825 |
| Caudate (basal ganglia) | rs12292980 | 0.305699 | 0.994845 | -0.2234 | 0.007318 | 0.052029 |
| Caudate (basal ganglia) | rs149967066 | 0.020619 | 1 | 0.2184 | 0.008773 | 0.060729 |
| Caudate (basal ganglia) | rs117853517 | 0.020619 | 1 | 0.2184 | 0.008773 | 0.060729 |
| Caudate (basal ganglia) | rs11030121 | 0.307292 | 0.989691 | -0.2145 | 0.010375 | 0.070071 |
| Caudate (basal ganglia) | rs1491850 | 0.417526 | 1 | 0.2104 | 0.011657 | 0.076604 |
| Caudate (basal ganglia) | rs11030119 | 0.283505 | 1 | -0.2044 | 0.014358 | 0.090627 |
| Caudate (basal ganglia) | rs7124442 | 0.702073 | 0.994845 | 0.2038 | 0.014625 | 0.091987 |
| Cerebellar Hemisphere | rs6265 | 0.162857 | 1 | 0.5475 | 9.10E-12 | 4.71E-10 |
| Cerebellar Hemisphere | rs12419948 | 0.177143 | 1 | 0.5367 | 2.74E-11 | 1.19E-09 |
| Cerebellar Hemisphere | rs925947 | 0.177143 | 1 | 0.5367 | 2.74E-11 | 1.19E-09 |
| Cerebellar Hemisphere | rs16917204 | 0.177143 | 1 | 0.5367 | 2.74E-11 | 1.19E-09 |
| Cerebellar Hemisphere | rs10501087 | 0.177143 | 1 | 0.5367 | 2.74E-11 | 1.19E-09 |
| Cerebellar Hemisphere | rs36070170 | 0.177143 | 1 | 0.5367 | 2.74E-11 | 1.19E-09 |
| Cerebellar Hemisphere | rs4923463 | 0.177143 | 1 | 0.5367 | 2.74E-11 | 1.19E-09 |
| Cerebellar Hemisphere | rs11030099 | 0.177143 | 1 | 0.5367 | 2.74E-11 | 1.19E-09 |
| Cerebellar Hemisphere | rs11030100 | 0.177143 | 1 | 0.5367 | 2.74E-11 | 1.19E-09 |
| Cerebellar Hemisphere | rs4923464 | 0.177143 | 1 | 0.5367 | 2.74E-11 | 1.19E-09 |
| Cerebellar Hemisphere | rs2049045 | 0.16 | 1 | 0.5266 | 7.51E-11 | 3.09E-09 |
| Cerebellar Hemisphere | rs11030104 | 0.174286 | 1 | 0.5254 | 8.45E-11 | 3.45E-09 |
| Cerebellar Hemisphere | rs1829469 | 0.16954 | 0.994286 | 0.5226 | 1.30E-10 | 5.27E-09 |
| Cerebellar Hemisphere | rs16917237 | 0.174286 | 1 | 0.5127 | 2.81E-10 | 9.48E-09 |
| Cerebellar Hemisphere | rs35038967 | 0.174286 | 1 | 0.5127 | 2.81E-10 | 9.48E-09 |
| Cerebellar Hemisphere | rs12575096 | 0.174286 | 1 | 0.5127 | 2.81E-10 | 9.48E-09 |
| Cerebellar Hemisphere | rs34379767 | 0.177143 | 1 | 0.4994 | 9.39E-10 | 3.05E-08 |
| Cerebellar Hemisphere | rs113145808 | 0.177143 | 1 | 0.4994 | 9.39E-10 | 3.05E-08 |
| Cerebellar Hemisphere | rs4923466 | 0.177143 | 1 | 0.4994 | 9.39E-10 | 3.05E-08 |
| Cerebellar Hemisphere | rs12801337 | 0.177143 | 1 | 0.4994 | 9.39E-10 | 3.05E-08 |
| Cerebellar Hemisphere | rs12790234 | 0.177143 | 1 | 0.4994 | 9.39E-10 | 3.05E-08 |
| Cerebellar Hemisphere | rs6484320 | 0.82 | 1 | -0.497 | 1.16E-09 | 3.68E-08 |
| Cerebellar Hemisphere | rs444654 | 0.82 | 1 | -0.497 | 1.16E-09 | 3.68E-08 |
| Cerebellar Hemisphere | rs7926362 | 0.82 | 1 | -0.497 | 1.16E-09 | 3.68E-08 |
| Cerebellar Hemisphere | rs7103411 | 0.822857 | 1 | -0.48 | 5.03E-09 | 1.51E-07 |
| Cerebellar Hemisphere | rs4922793 | 0.18 | 1 | 0.4782 | 5.83E-09 | 1.73E-07 |
| Cerebellar Hemisphere | rs988748 | 0.817143 | 1 | -0.476 | 7.02E-09 | 2.06E-07 |
| Cerebellar Hemisphere | rs10767664 | 0.817143 | 1 | -0.476 | 7.02E-09 | 2.06E-07 |
| Cerebellar Hemisphere | rs2030323 | 0.817143 | 1 | -0.476 | 7.02E-09 | 2.06E-07 |
| Cerebellar Hemisphere | rs71311904 | 0.174286 | 1 | 0.4562 | 3.43E-08 | 9.58E-07 |
| Cerebellar Hemisphere | rs10219241 | 0.468391 | 0.994286 | 0.4 | 2.01E-06 | 4.49E-05 |
| Cerebellar Hemisphere | rs7931247 | 0.528571 | 1 | -0.3945 | 2.62E-06 | 5.56E-05 |
| Cerebellar Hemisphere | rs2030324 | 0.525714 | 1 | -0.383 | 5.38E-06 | 0.000104 |
| Cerebellar Hemisphere | rs7934165 | 0.525714 | 1 | -0.383 | 5.38E-06 | 0.000104 |
| Cerebellar Hemisphere | rs10767665 | 0.525714 | 1 | -0.383 | 5.38E-06 | 0.000104 |
| Cerebellar Hemisphere | rs10767667 | 0.525714 | 1 | -0.383 | 5.38E-06 | 0.000104 |
| Cerebellar Hemisphere | rs10835215 | 0.523256 | 0.982857 | -0.3809 | 7.78E-06 | 0.000147 |
| Cerebellar Hemisphere | rs4378341 | 0.517442 | 0.982857 | -0.3778 | 8.65E-06 | 0.00016 |
| Cerebellar Hemisphere | rs7103873 | 0.52 | 1 | -0.3727 | 1.00E-05 | 0.000183 |
| Cerebellar Hemisphere | rs7104207 | 0.52 | 1 | -0.3727 | 1.00E-05 | 0.000183 |
| Cerebellar Hemisphere | rs4633417 | 0.52 | 1 | -0.3727 | 1.00E-05 | 0.000183 |
| Cerebellar Hemisphere | rs7482752 | 0.517241 | 0.994286 | -0.3727 | 1.00E-05 | 0.000183 |
| Cerebellar Hemisphere | rs371590941 | 0.511628 | 0.982857 | -0.3655 | 1.63E-05 | 0.000294 |
| Cerebellar Hemisphere | rs753875000 | 0.508824 | 0.971429 | -0.3662 | 1.69E-05 | 0.000303 |
| Cerebellar Hemisphere | rs1491193668 | 0.508824 | 0.971429 | -0.3662 | 1.69E-05 | 0.000303 |
| Cerebellar Hemisphere | rs2049046 | 0.517143 | 1 | -0.3609 | 1.98E-05 | 0.000351 |
| Cerebellar Hemisphere | rs10767662 | 0.517341 | 0.988571 | -0.3606 | 2.33E-05 | 0.000403 |
| Cerebellar Hemisphere | rs4385847 | 0.517241 | 0.994286 | -0.3572 | 2.61E-05 | 0.00045 |
| Cerebellar Hemisphere | rs4542361 | 0.514451 | 0.988571 | -0.3429 | 6.10E-05 | 0.001002 |
| Cerebellar Hemisphere | rs2883187 | 0.491429 | 1 | -0.3372 | 7.23E-05 | 0.00117 |
| Cerebellar Hemisphere | rs1519479 | 0.494286 | 1 | -0.3084 | 0.000305 | 0.004568 |
| Cerebellar Hemisphere | rs3045304 | 0.494286 | 1 | -0.3084 | 0.000305 | 0.004568 |
| Cerebellar Hemisphere | rs2203877 | 0.491429 | 1 | -0.3022 | 0.000408 | 0.005985 |
| Cerebellar Hemisphere | rs11030101 | 0.479885 | 0.994286 | -0.2997 | 0.000481 | 0.006978 |
| Cerebellar Hemisphere | rs1491850 | 0.405714 | 1 | 0.2945 | 0.00058 | 0.008284 |
| Cerebellar Hemisphere | rs369811230 | 0.485549 | 0.988571 | -0.293 | 0.000652 | 0.009028 |
| Cerebellar Hemisphere | rs10835210 | 0.451429 | 1 | -0.2805 | 0.001073 | 0.013759 |
| Cerebellar Hemisphere | rs1949513 | 0.451429 | 1 | -0.2805 | 0.001073 | 0.013759 |
| Cerebellum | rs6265 | 0.155502 | 1 | 0.584 | 4.12E-17 | 3.00E-15 |
| Cerebellum | rs2049045 | 0.15311 | 1 | 0.5676 | 4.73E-16 | 2.95E-14 |
| Cerebellum | rs12419948 | 0.174641 | 1 | 0.5518 | 4.31E-15 | 2.27E-13 |
| Cerebellum | rs925947 | 0.174641 | 1 | 0.5518 | 4.31E-15 | 2.27E-13 |
| Cerebellum | rs16917204 | 0.174641 | 1 | 0.5518 | 4.31E-15 | 2.27E-13 |
| Cerebellum | rs10501087 | 0.174641 | 1 | 0.5518 | 4.31E-15 | 2.27E-13 |
| Cerebellum | rs36070170 | 0.174641 | 1 | 0.5518 | 4.31E-15 | 2.27E-13 |
| Cerebellum | rs4923463 | 0.174641 | 1 | 0.5518 | 4.31E-15 | 2.27E-13 |
| Cerebellum | rs11030099 | 0.174641 | 1 | 0.5518 | 4.31E-15 | 2.27E-13 |
| Cerebellum | rs11030100 | 0.174641 | 1 | 0.5518 | 4.31E-15 | 2.27E-13 |
| Cerebellum | rs4923464 | 0.174641 | 1 | 0.5518 | 4.31E-15 | 2.27E-13 |
| Cerebellum | rs11030104 | 0.172249 | 1 | 0.5452 | 1.05E-14 | 4.95E-13 |
| Cerebellum | rs1829469 | 0.167476 | 0.985646 | 0.5391 | 3.97E-14 | 1.76E-12 |
| Cerebellum | rs16917237 | 0.169856 | 1 | 0.5185 | 3.21E-13 | 1.26E-11 |
| Cerebellum | rs35038967 | 0.169856 | 1 | 0.5185 | 3.21E-13 | 1.26E-11 |
| Cerebellum | rs12575096 | 0.169856 | 1 | 0.5185 | 3.21E-13 | 1.26E-11 |
| Cerebellum | rs6484320 | 0.820574 | 1 | -0.514 | 5.51E-13 | 2.06E-11 |
| Cerebellum | rs444654 | 0.820574 | 1 | -0.514 | 5.51E-13 | 2.06E-11 |
| Cerebellum | rs7103411 | 0.822967 | 1 | -0.5098 | 9.16E-13 | 3.39E-11 |
| Cerebellum | rs7926362 | 0.822115 | 0.995215 | -0.5099 | 1.06E-12 | 3.88E-11 |
| Cerebellum | rs34379767 | 0.173077 | 0.995215 | 0.5075 | 1.20E-12 | 4.24E-11 |
| Cerebellum | rs113145808 | 0.174641 | 1 | 0.5075 | 1.20E-12 | 4.24E-11 |
| Cerebellum | rs12801337 | 0.174641 | 1 | 0.5075 | 1.20E-12 | 4.24E-11 |
| Cerebellum | rs12790234 | 0.174641 | 1 | 0.5075 | 1.20E-12 | 4.24E-11 |
| Cerebellum | rs4923466 | 0.173077 | 0.995215 | 0.5086 | 1.22E-12 | 4.25E-11 |
| Cerebellum | rs4922793 | 0.175481 | 0.995215 | 0.5019 | 2.69E-12 | 9.02E-11 |
| Cerebellum | rs988748 | 0.818182 | 1 | -0.4983 | 3.49E-12 | 1.09E-10 |
| Cerebellum | rs10767664 | 0.818182 | 1 | -0.4983 | 3.49E-12 | 1.09E-10 |
| Cerebellum | rs2030323 | 0.818182 | 1 | -0.4983 | 3.49E-12 | 1.09E-10 |
| Cerebellum | rs71311904 | 0.172249 | 1 | 0.4743 | 4.96E-11 | 1.49E-09 |
| Cerebellum | rs7931247 | 0.526316 | 1 | -0.3858 | 1.73E-07 | 3.66E-06 |
| Cerebellum | rs2030324 | 0.523923 | 1 | -0.3726 | 4.81E-07 | 9.61E-06 |
| Cerebellum | rs7934165 | 0.523923 | 1 | -0.3726 | 4.81E-07 | 9.61E-06 |
| Cerebellum | rs10767665 | 0.523923 | 1 | -0.3726 | 4.81E-07 | 9.61E-06 |
| Cerebellum | rs10767667 | 0.523923 | 1 | -0.3726 | 4.81E-07 | 9.61E-06 |
| Cerebellum | rs10835215 | 0.521739 | 0.990431 | -0.3745 | 4.87E-07 | 9.65E-06 |
| Cerebellum | rs10219241 | 0.471154 | 0.995215 | 0.3723 | 5.34E-07 | 1.05E-05 |
| Cerebellum | rs10767662 | 0.512077 | 0.990431 | -0.3624 | 1.11E-06 | 2.12E-05 |
| Cerebellum | rs4378341 | 0.512136 | 0.985646 | -0.3631 | 1.14E-06 | 2.17E-05 |
| Cerebellum | rs7103873 | 0.514354 | 1 | -0.3596 | 1.27E-06 | 2.37E-05 |
| Cerebellum | rs7104207 | 0.514354 | 1 | -0.3596 | 1.27E-06 | 2.37E-05 |
| Cerebellum | rs4633417 | 0.514354 | 1 | -0.3596 | 1.27E-06 | 2.37E-05 |
| Cerebellum | rs7482752 | 0.512019 | 0.995215 | -0.3596 | 1.27E-06 | 2.37E-05 |
| Cerebellum | rs2049046 | 0.511962 | 1 | -0.3513 | 2.31E-06 | 4.12E-05 |
| Cerebellum | rs4542361 | 0.509662 | 0.990431 | -0.3494 | 2.81E-06 | 4.82E-05 |
| Cerebellum | rs4385847 | 0.512019 | 0.995215 | -0.3494 | 2.81E-06 | 4.82E-05 |
| Cerebellum | rs371590941 | 0.504902 | 0.976077 | -0.3518 | 2.92E-06 | 4.91E-05 |
| Cerebellum | rs753875000 | 0.502475 | 0.966507 | -0.3523 | 3.02E-06 | 5.04E-05 |
| Cerebellum | rs1491193668 | 0.502475 | 0.966507 | -0.3523 | 3.02E-06 | 5.04E-05 |
| Cerebellum | rs1491850 | 0.399522 | 1 | 0.3321 | 8.58E-06 | 0.000123 |
| Cerebellum | rs1491851 | 0.466507 | 1 | 0.3267 | 1.22E-05 | 0.000166 |
| Cerebellum | rs1519479 | 0.495215 | 1 | -0.3206 | 1.80E-05 | 0.00024 |
| Cerebellum | rs3045304 | 0.495215 | 1 | -0.3206 | 1.80E-05 | 0.00024 |
| Cerebellum | rs2203877 | 0.490431 | 1 | -0.3158 | 2.44E-05 | 0.000317 |
| Cerebellum | rs2883187 | 0.495215 | 1 | -0.3108 | 3.34E-05 | 0.000427 |
| Cerebellum | rs7481773 | 0.421053 | 1 | 0.2968 | 7.70E-05 | 0.00095 |
| Cerebellum | rs11030101 | 0.478365 | 0.995215 | -0.2923 | 0.000105 | 0.001269 |
| Cerebellum | rs369811230 | 0.485507 | 0.990431 | -0.2816 | 0.000191 | 0.002268 |
| Cerebellum | rs10835210 | 0.447368 | 1 | -0.2793 | 0.000207 | 0.002457 |
| Cerebellum | rs1949513 | 0.449519 | 0.995215 | -0.2703 | 0.00035 | 0.004032 |
| Cerebellum | rs75298795 | 0.126794 | 1 | -0.1961 | 0.009923 | 0.080676 |
| Cortex | rs6265 | 0.139024 | 1 | 0.5974 | 1.10E-15 | 2.39E-13 |
| Cortex | rs2049045 | 0.136585 | 1 | 0.5758 | 1.93E-14 | 3.46E-12 |
| Cortex | rs12419948 | 0.158537 | 1 | 0.5069 | 4.90E-11 | 3.53E-09 |
| Cortex | rs925947 | 0.158537 | 1 | 0.5069 | 4.90E-11 | 3.53E-09 |
| Cortex | rs16917204 | 0.158537 | 1 | 0.5069 | 4.90E-11 | 3.53E-09 |
| Cortex | rs10501087 | 0.158537 | 1 | 0.5069 | 4.90E-11 | 3.53E-09 |
| Cortex | rs36070170 | 0.158537 | 1 | 0.5069 | 4.90E-11 | 3.53E-09 |
| Cortex | rs4923463 | 0.158537 | 1 | 0.5069 | 4.90E-11 | 3.53E-09 |
| Cortex | rs11030099 | 0.158537 | 1 | 0.5069 | 4.90E-11 | 3.53E-09 |
| Cortex | rs11030100 | 0.158537 | 1 | 0.5069 | 4.90E-11 | 3.53E-09 |
| Cortex | rs4923464 | 0.158537 | 1 | 0.5069 | 4.90E-11 | 3.53E-09 |
| Cortex | rs11030104 | 0.156098 | 1 | 0.4978 | 1.21E-10 | 8.18E-09 |
| Cortex | rs4922793 | 0.159314 | 0.995122 | 0.4918 | 2.53E-10 | 1.60E-08 |
| Cortex | rs7103411 | 0.839024 | 1 | -0.4896 | 2.71E-10 | 1.63E-08 |
| Cortex | rs6484320 | 0.839024 | 1 | -0.4896 | 2.71E-10 | 1.63E-08 |
| Cortex | rs444654 | 0.839024 | 1 | -0.4896 | 2.71E-10 | 1.63E-08 |
| Cortex | rs7926362 | 0.839024 | 1 | -0.4896 | 2.71E-10 | 1.63E-08 |
| Cortex | rs988748 | 0.834146 | 1 | -0.4896 | 2.71E-10 | 1.63E-08 |
| Cortex | rs10767664 | 0.836585 | 1 | -0.4896 | 2.71E-10 | 1.63E-08 |
| Cortex | rs2030323 | 0.836585 | 1 | -0.4896 | 2.71E-10 | 1.63E-08 |
| Cortex | rs4923466 | 0.156863 | 0.995122 | 0.4866 | 4.13E-10 | 2.38E-08 |
| Cortex | rs16917237 | 0.153659 | 1 | 0.4789 | 7.42E-10 | 4.18E-08 |
| Cortex | rs35038967 | 0.153659 | 1 | 0.4789 | 7.42E-10 | 4.18E-08 |
| Cortex | rs12575096 | 0.153659 | 1 | 0.4789 | 7.42E-10 | 4.18E-08 |
| Cortex | rs113145808 | 0.158537 | 1 | 0.4758 | 9.88E-10 | 5.40E-08 |
| Cortex | rs12801337 | 0.158537 | 1 | 0.4758 | 9.88E-10 | 5.40E-08 |
| Cortex | rs12790234 | 0.158537 | 1 | 0.4758 | 9.88E-10 | 5.40E-08 |
| Cortex | rs34379767 | 0.156863 | 0.995122 | 0.476 | 1.11E-09 | 5.94E-08 |
| Cortex | rs1829469 | 0.15099 | 0.985366 | 0.4754 | 1.17E-09 | 6.20E-08 |
| Cortex | rs71311904 | 0.156098 | 1 | 0.455 | 6.28E-09 | 3.10E-07 |
| Cortex | rs7481773 | 0.42439 | 1 | 0.3168 | 8.73E-05 | 0.001617 |
| Cortex | rs1491851 | 0.470732 | 1 | 0.2985 | 0.000229 | 0.003518 |
| Cortex | rs1491850 | 0.365854 | 1 | 0.2458 | 0.002604 | 0.021076 |
| Cortex | rs10219241 | 0.441463 | 1 | 0.2363 | 0.003836 | 0.029393 |
| Cortex | rs10767659 | 0.659314 | 0.995122 | 0.2345 | 0.004253 | 0.03207 |
| Cortex | rs78500263 | 0.658537 | 1 | 0.2333 | 0.004322 | 0.032499 |
| Cortex | rs4517468 | 0.658537 | 1 | 0.2333 | 0.004322 | 0.032499 |
| Cortex | rs2353487 | 0.656863 | 0.995122 | 0.2333 | 0.004322 | 0.032499 |
| Cortex | rs2030324 | 0.55122 | 1 | -0.2253 | 0.005903 | 0.04178 |
| Cortex | rs7934165 | 0.55122 | 1 | -0.2253 | 0.005903 | 0.04178 |
| Cortex | rs10767665 | 0.55122 | 1 | -0.2253 | 0.005903 | 0.04178 |
| Cortex | rs10767667 | 0.55122 | 1 | -0.2253 | 0.005903 | 0.04178 |
| Cortex | rs7931247 | 0.553659 | 1 | -0.2253 | 0.005903 | 0.04178 |
| Cortex | rs10835215 | 0.549505 | 0.985366 | -0.2259 | 0.006291 | 0.043346 |
| Cortex | rs76069707 | 0.652709 | 0.990244 | 0.2228 | 0.006492 | 0.043973 |
| Cortex | rs10767662 | 0.536946 | 0.990244 | -0.2076 | 0.011648 | 0.069463 |
| Cortex | rs4378341 | 0.537129 | 0.985366 | -0.2051 | 0.013033 | 0.076402 |
| Cortex | rs7103873 | 0.539024 | 1 | -0.2034 | 0.013177 | 0.076896 |
| Cortex | rs7104207 | 0.539024 | 1 | -0.2034 | 0.013177 | 0.076896 |
| Cortex | rs4633417 | 0.539024 | 1 | -0.2034 | 0.013177 | 0.076896 |
| Cortex | rs4542361 | 0.534483 | 0.990244 | -0.2034 | 0.013177 | 0.076896 |
| Cortex | rs4385847 | 0.536765 | 0.995122 | -0.2034 | 0.013177 | 0.076896 |
| Cortex | rs7482752 | 0.539024 | 1 | -0.2034 | 0.013177 | 0.076896 |
| Cortex | rs2049046 | 0.536585 | 1 | -0.2034 | 0.013177 | 0.076896 |
| Cortex | rs1519480 | 0.670732 | 1 | 0.2006 | 0.014523 | 0.083625 |
| Nucleus accumbens (basal ganglia) | rs2049045 | 0.163366 | 1 | 0.4088 | 3.02E-07 | 3.04E-05 |
| Nucleus accumbens (basal ganglia) | rs6265 | 0.165842 | 1 | 0.4084 | 3.09E-07 | 3.05E-05 |
| Nucleus accumbens (basal ganglia) | rs7103411 | 0.819307 | 1 | -0.3847 | 1.63E-06 | 9.15E-05 |
| Nucleus accumbens (basal ganglia) | rs7926362 | 0.818408 | 0.99505 | -0.3768 | 3.00E-06 | 0.000143 |
| Nucleus accumbens (basal ganglia) | rs6484320 | 0.816832 | 1 | -0.3755 | 3.02E-06 | 0.000143 |
| Nucleus accumbens (basal ganglia) | rs444654 | 0.816832 | 1 | -0.3755 | 3.02E-06 | 0.000143 |
| Nucleus accumbens (basal ganglia) | rs988748 | 0.816832 | 1 | -0.3755 | 3.02E-06 | 0.000143 |
| Nucleus accumbens (basal ganglia) | rs10767664 | 0.816832 | 1 | -0.3755 | 3.02E-06 | 0.000143 |
| Nucleus accumbens (basal ganglia) | rs2030323 | 0.816832 | 1 | -0.3755 | 3.02E-06 | 0.000143 |
| Nucleus accumbens (basal ganglia) | rs34379767 | 0.178218 | 1 | 0.3694 | 4.47E-06 | 0.000193 |
| Nucleus accumbens (basal ganglia) | rs113145808 | 0.178218 | 1 | 0.3694 | 4.47E-06 | 0.000193 |
| Nucleus accumbens (basal ganglia) | rs4923466 | 0.178218 | 1 | 0.3694 | 4.47E-06 | 0.000193 |
| Nucleus accumbens (basal ganglia) | rs12801337 | 0.178218 | 1 | 0.3694 | 4.47E-06 | 0.000193 |
| Nucleus accumbens (basal ganglia) | rs12790234 | 0.178218 | 1 | 0.3694 | 4.47E-06 | 0.000193 |
| Nucleus accumbens (basal ganglia) | rs4922793 | 0.176617 | 0.99505 | 0.3694 | 4.47E-06 | 0.000193 |
| Nucleus accumbens (basal ganglia) | rs12419948 | 0.180693 | 1 | 0.3672 | 5.12E-06 | 0.000198 |
| Nucleus accumbens (basal ganglia) | rs925947 | 0.180693 | 1 | 0.3672 | 5.12E-06 | 0.000198 |
| Nucleus accumbens (basal ganglia) | rs16917204 | 0.180693 | 1 | 0.3672 | 5.12E-06 | 0.000198 |
| Nucleus accumbens (basal ganglia) | rs10501087 | 0.180693 | 1 | 0.3672 | 5.12E-06 | 0.000198 |
| Nucleus accumbens (basal ganglia) | rs36070170 | 0.180693 | 1 | 0.3672 | 5.12E-06 | 0.000198 |
| Nucleus accumbens (basal ganglia) | rs4923463 | 0.180693 | 1 | 0.3672 | 5.12E-06 | 0.000198 |
| Nucleus accumbens (basal ganglia) | rs11030099 | 0.180693 | 1 | 0.3672 | 5.12E-06 | 0.000198 |
| Nucleus accumbens (basal ganglia) | rs11030100 | 0.180693 | 1 | 0.3672 | 5.12E-06 | 0.000198 |
| Nucleus accumbens (basal ganglia) | rs4923464 | 0.180693 | 1 | 0.3672 | 5.12E-06 | 0.000198 |
| Nucleus accumbens (basal ganglia) | rs11030104 | 0.180693 | 1 | 0.3672 | 5.12E-06 | 0.000198 |
| Nucleus accumbens (basal ganglia) | rs1829469 | 0.17 | 0.990099 | 0.3581 | 9.10E-06 | 0.000288 |
| Nucleus accumbens (basal ganglia) | rs71311904 | 0.173267 | 1 | 0.3561 | 1.03E-05 | 0.000315 |
| Nucleus accumbens (basal ganglia) | rs16917237 | 0.175743 | 1 | 0.3506 | 1.44E-05 | 0.000422 |
| Nucleus accumbens (basal ganglia) | rs35038967 | 0.175743 | 1 | 0.3506 | 1.44E-05 | 0.000422 |
| Nucleus accumbens (basal ganglia) | rs12575096 | 0.175743 | 1 | 0.3506 | 1.44E-05 | 0.000422 |
| Nucleus accumbens (basal ganglia) | rs2353487 | 0.649254 | 0.99505 | 0.3028 | 0.000213 | 0.00341 |
| Nucleus accumbens (basal ganglia) | rs76069707 | 0.641791 | 0.99505 | 0.2961 | 0.000285 | 0.004349 |
| Nucleus accumbens (basal ganglia) | rs1519480 | 0.660891 | 1 | 0.2924 | 0.000341 | 0.005092 |
| Nucleus accumbens (basal ganglia) | rs78500263 | 0.65099 | 1 | 0.2899 | 0.000387 | 0.005612 |
| Nucleus accumbens (basal ganglia) | rs10767659 | 0.65099 | 1 | 0.2899 | 0.000387 | 0.005612 |
| Nucleus accumbens (basal ganglia) | rs4517468 | 0.654229 | 0.99505 | 0.2899 | 0.000387 | 0.005612 |
| Nucleus accumbens (basal ganglia) | rs7127507 | 0.305 | 0.990099 | -0.238 | 0.004077 | 0.040738 |
| Nucleus accumbens (basal ganglia) | rs1808124 | 0.690594 | 1 | 0.2314 | 0.004943 | 0.045862 |
| Nucleus accumbens (basal ganglia) | rs727155 | 0.034653 | 1 | -0.222 | 0.007082 | 0.055666 |
| Nucleus accumbens (basal ganglia) | rs7124442 | 0.706468 | 0.99505 | 0.2216 | 0.007189 | 0.056235 |
| Nucleus accumbens (basal ganglia) | rs10767658 | 0.728856 | 0.99505 | 0.2164 | 0.008931 | 0.066095 |
| Nucleus accumbens (basal ganglia) | rs185690171 | 0.019802 | 1 | -0.2078 | 0.01186 | 0.080914 |
| Nucleus accumbens (basal ganglia) | rs17309874 | 0.232673 | 1 | -0.2076 | 0.011944 | 0.080914 |
| Nucleus accumbens (basal ganglia) | rs925946 | 0.727723 | 1 | 0.2069 | 0.012241 | 0.080991 |
| Nucleus accumbens (basal ganglia) | rs1401635 | 0.717822 | 1 | 0.1995 | 0.015764 | 0.097901 |
| Nucleus accumbens (basal ganglia) | rs11030108 | 0.717822 | 1 | 0.1995 | 0.015764 | 0.097901 |
| Nucleus accumbens (basal ganglia) | rs149967066 | 0.019802 | 1 | 0.1994 | 0.015804 | 0.097901 |
| Nucleus accumbens (basal ganglia) | rs117853517 | 0.019802 | 1 | 0.1994 | 0.015804 | 0.097901 |
